# Solid Dispersions of Genistein via Solvent Rotary Evaporation for Improving Solubility, Bioavailability, and Amelioration Effect in HFD-Induced Obesity Mice

**DOI:** 10.3390/pharmaceutics16030306

**Published:** 2024-02-22

**Authors:** Chenxu Qiu, Yancui Zhang, Yingsai Fan, Shupeng Li, Jianting Gao, Xin He, Xinghua Zhao

**Affiliations:** College of Veterinary Medicine, Hebei Agricultural University, Baoding 071000, China; chenxu235811@163.com (C.Q.); zyc15531218962@163.com (Y.Z.); fanyingsai@126.com (Y.F.); lspeng2005@163.com (S.L.); gaojianting9908@163.com (J.G.); dyhexin@hebau.edu.cn (X.H.)

**Keywords:** genistein, solid dispersion, dissolution rate, bioavailability, obesity

## Abstract

Genistein (GEN) is an active pharmaceutical ingredient that presents the challenges of poor water solubility and low oral bioavailability. To tackle these challenges, a GEN solid dispersion was prepared by solvent rotary evaporation using polyvinylpyrrolidone K30 (PVP K30) as a carrier. The optimal formulation was determined by drug loading efficiency and in vitro release. The physical state of the solid dispersion was characterized by DSC, XRD, SEM and FT-IR. And the results of the in vitro release study indicate that the drug release of SD (1:7) increased 482-fold that of pure GEN at 60 min. Following oral administration to rats, the C_max_ and AUC_0–24_ of SD (1:7) was increased 6.86- and 2.06-fold to that of pure GEN. The adipose fat index and body weight of the SD (1:7) group were significantly lower than those of the GEN group (*p* < 0.05). Meanwhile, the levels of TC and TG in the serum were significantly decreased in the SD (1:7) group compared with the GEN group (*p* < 0.05). All experiments revealed that solid dispersion could be a promising formulation approach to improve the dissolution rate, oral bioavailability, and effect on the reduction of lipid accumulation in high-fat diet-induced obesity mice.

## 1. Introduction

**Figure 1:**
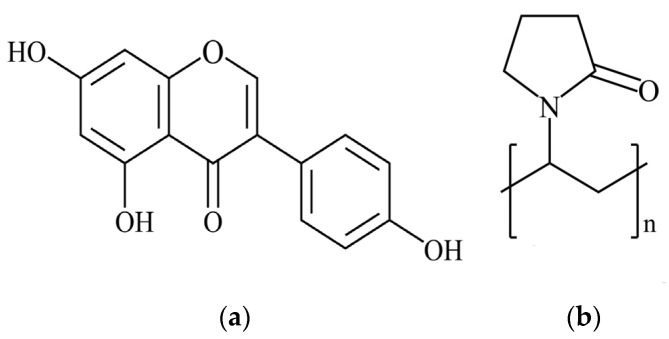
Chemical structures of (**a**) GEN and (**b**) PVP K30.

Genistein (4′,5,7-trihydroxyisoflavone, GEN, Figure 1a), the most abundant isoflavonoid in traditional Chinese medicines such as *kudzuvine root* [1], soybean, and, especially, fermented soybean products [2,3], has recently received considerable attention for the treatment of lipid metabolic disorders [4,5,6] and reducing the effects of obesity [7]. However, as a Biopharmaceutics Classification System (BCS) class II drug, the poor aqueous solubility (0.029 mg/mL) of GEN causes low bioavailability, limiting its use in pure form [8,9,10]. Various methods have been developed to improve the low solubility of GEN, including micelles [11], nanoparticles [12], solid-lipid nanoparticles [13], microemulsions [14], cocrystals [1], and solid dispersions (SDs) [15,16]. Of these methods, an SD is formed by incorporating the drug uniformly in a highly dispersed state in a solid carrier and has the advantages of simplicity, convenience, and efficiency [17,18]. Genistein solid dispersion has been successfully produced by the hot-melt extrusion method [16]. In SD systems, the solubility of insoluble drugs can be improved by reducing particle size and enhancing wettability and dispersibility by the formation of an amorphous state [19,20]. While the in vitro solubility of these was significantly improved, the SD absorption in vivo has not been studied. Polyvinylpyrrolidone (PVP) is a commonly used carrier material with the advantages of non-toxicity, pH stability, temperature resistance, and good biocompatibility [21]. It is widely used in the pharmaceutical industry, food industry, cosmetics, and other industries. Studies have shown that PVP is a safe pharmaceutical excipient and food additive and that the daily intake proposed by WHO is 0–50 mg/kg weight. The K value of PVP indicates its average molecular weight, and the greater the K value, the higher the viscosity and the better the solubilization effect [22]. However, a high K value will lead to the excessive viscosity of the SD while a low K value cannot achieve the expected solubilization effect. So, PVP K30 (Figure 1b) was chosen as the carrier material. An alternative SD preparation method by solvent rotary evaporation has the advantages of a simple operation and low cost [23]. The solvent can be removed at a lower temperature, helping prevent the decomposition of the drug or carrier [24]. In this work, GEN SD was successfully synthesized by solvent rotary evaporation using PVP K30 as a carrier. The SD characterization, dissolution behavior, pharmacokinetic properties, and stability, and its effect on serum lipid levels, the liver index, and the histopathological examination of HFD-induced obese mice were investigated.

## 2. Materials and Methods

### 2.1. Materials

GEN (purity ≥ 98%) was acquired from Biopurify Phytochemicals Ltd. (Chengdu, China). GEN standard (purity ≥ 99.5%) was purchased from the National Institute for Food and Drug Control (Beijing, China). PVP K30 was obtained from Shanghai Aladdin Bio-Chem Technology Co., Ltd. (Shanghai, China). Chromatography-grade methanol was provided by Thermo Fisher Scientific Inc. (Shanghai, China) and other chemicals were obtained from commercial suppliers and used directly.

Male Sprague-Dawley rats (240–260 g in weight, 8–9 weeks of age) and male ICR mice (15–20 g in weight, 5–6 weeks of age) were supplied by SPF Biotechnology Co., Ltd. (Beijing, China). The animals were kept in a controlled environment (temperature and humidity). The use of animals in this study was approved by the Institutional Animal Care and Ethical Committee of Hebei Agricultural University.

### 2.2. Preparation of GEN SDs and Physical Mixtures (PMs)

GEN SDs were prepared by the solvent rotary evaporation method. GEN and PVP K30 were weighed in ratios of 1:1, 1:3, 1:5, 1:7, and 1:9 (*w*/*w*), respectively, and then dissolved in ethanol and evaporated to dryness using a rotary evaporator (RE-52AA, YaRong, Shanghai, China) on a water bath at 45 °C at a pressure of −0.01 MPa. The materials after rotary evaporation (denoted SD (1:1–9)) were dried in a fume hood at 25 °C to remove the residual solvent.

The PMs of GEN and PVP K30 in the same ratios as above (PM (1:1–9)) were made by grinding with a vortex meter (Vortex-5, Kylin-Bell Ltd., Haimen, China). 

The SDs and PMs were milled through a 100-mesh sieve (75–100 μm) and stored in a desiccator for further use.

### 2.3. Powder X-ray Diffraction (PXRD) and Differential Scanning Calorimetry (DSC)

An X-ray diffractometer (D8 Advance, Bruker, Billerica, MA, USA) was used to obtain the PXRD patterns of pure GEN, polymer PVP K30, SDs, and PMs. Samples were scanned over the 2θ range of 5 to 35°, with a 0.01° step size and a residence time for each step of 0.1 s while using a radiation source (Cu-Ka, λ = 1.5418 Å) at 15 mA and 40 kV.

A differential scanning calorimeter (Q2000, TA Instruments, New Castle, DE, USA) was used to study the thermal properties of the samples. Powders (3–5 mg) of pure GEN, polymer PVP K30, SDs, and PMs were placed in an aluminum crucible with holes punched in the crucible lid in advance. The measurement was performed at a heating from 25 to 320 °C under a nitrogen purge of 50 mL/min rate with a heating rate of 10 °C /min.

### 2.4. High-Performance Liquid Chromatography (HPLC) Analysis

The concentration of GEN was measured by a model 1525 HPLC system (Waters Corporation, Milford, MA, USA) with a model 2998 photodiode array (PDA) detector (Waters, Milford MA, USA) and a C18 reversed phase column (5 μm, 250 mm × 4.6 mm). For in vitro dissolution testing and pharmacokinetics studies, the mobile phase was composed of methanol–water (pH adjusted to 2.4 with phosphoric acid) at a ratio of 70:30 and 60:40 (*v*/*v*, respectively). The flow rate was 1.0 mL/min. The column temperature was maintained at 37 °C and the UV detection wavelength was 261 nm.

### 2.5. Drug Loading Efficiency of SDs

Approximately 20 mg of SDs was dissolved in 5 mL of ethanol. Then, the concentration of GEN was measured using the UV spectrophotometer 6850 (Jenway, Chicago, IL, USA). The calculation was performed using Equation (1) [25].

(1) $$\mathrm{Drug}\;\mathrm{loading}\%\;=\;\frac{\mathrm{Weight}\;\mathrm{of}\;\mathrm{drug}\;\mathrm{in}\;\mathrm{SD}}{\mathrm{Weight}\;\mathrm{of}\;\mathrm{SD}}\;\times \;100$$

### 2.6. Saturation Solubility and In Vitro Dissolution Testing

The solubility of GEN in SDs with pH 6.8 phosphate buffer was measured using a shake-flask method. An excess amount of GEN was added to the above solutions, which were transferred to an air bath thermostatic shaker at 37 °C for 24 h. The solution was filtered through a 0.22 µm membrane filter [26]. The GEN concentration was measured at λ_max_ 261 nm using a UV spectrophotometer 6850 (Jenway, Chicago, IL, USA).

The dissolution tests were performed by a D-800LS dissolution tester (Tianjin, China) produced by the Precision Instrument Factory of Tianjin University. Excess pure GEN, SD, and PM samples (containing 100 mg of GEN) were placed in a phosphate buffer solution (900 mL, pH 6.8). The test mixture was maintained at 37 ± 0.5 °C with the paddle speed at 250 rpm [1]. An aliquot of the dissolution medium was withdrawn at 2, 5, 10, 20, 30, 60, 90, 120, 240, 360, and 480 min. An equal volume of temperature-equilibrated blank medium was then added to the beaker. The samples were filtered using a 0.22 µm filter, diluted to the appropriate concentration, and the drug concentration was analyzed by HPLC. Each sample was measured in triplicate.

### 2.7. Fourier Transform Infrared Spectroscopy (FT-IR)

The Fourier transform infrared spectra of pure GEN, PVP K30, SD (1:7), and PM (1:7) were collected using an infrared spectrometer (Nicolet iS5, ThermoFisher, Waltham, MA, USA). The samples (2 mg) were mixed with KBr (200 mg), applying a pressure of 10 MPa for 1 min to compress into pellets. The scanning range was 4000 to 500 cm^−1^, 64 scans per sample for collection, with a resolution of 2 cm^−1^.

### 2.8. Scanning Electron Microscopy (SEM)

The morphologies of samples GEN, PVP K30, SD (1:7), and PM (1:7) were characterized using a SEM (Hitachi, S4800, Tokyo, Japan). Prior to examination, the samples were directly dispersed on an electrically conductive adhesive tape and made conductive by gold coating. The difference between the sample powders was observed at an excitation voltage of 5 kV.

### 2.9. Physical Stability Studies

To assess the physical stability, SDs (1:7) were placed into open glass vials and exposed to 40 °C/75% relative humidity (RH) for 180 days. The samples were subsequently analyzed by PXRD at days 0, 15, 30, 60, 90, and 180.

### 2.10. In Vivo Pharmacokinetic Studies

Healthy Sprague-Dawley rats had free access to food and water until 12 h before the experiments, when they were randomly divided into two groups to receive pure GEN or SD, prepared by dispersing the powders in peanut oil at a dose of 50 mg GEN/kg. About 500 μL of blood samples were collected from the orbital sinus at 0.083, 0.25, 0.5, 0.75, 1, 2, 3, 4, 6, 8, 12, and 24 h after administration. Normal heparin was used as an anticoagulant. Blood samples were centrifuged at 6000 rpm for 10 min, and the plasma samples were stored at −20 °C until further analysis. The plasma samples (100 μL) were then mixed with acetonitrile (200 μL). The denatured protein precipitate was separated by vortex for 120 s and centrifuged at 12,000 rpm for 10 min. The supernatant was collected and filtered with a 0.22 μm filter membrane prior to HPLC analysis.

The maximal plasma concentration (C_max_), the time to reach maximum concentration (T_max_), the half-life (t_1/2_), and the area under the plasma concentration time curve (AUC_0−t_) were calculated by analyzing the plasma concentration time profile using a noncompartmental model with DAS 2.0 (Mathematical Pharmacology Professional Committee of China, Shanghai, China).

### 2.11. Pharmacodynamic Studies

#### 2.11.1. Preparation of High-Fat Model Mice

After one week of adaptation, the mice were randomly divided into 2 groups. The mice of the normal control (NC) group (*n* = 10) were fed a standard diet (365 kcal per 100 g feed) provided by SPF Biotechnology Co., Ltd. (Beijing, China). The remaining mice were fed a high-fat diet consisting of 55% standard commercial laboratory diet, 20% lard, 10% egg yolk power, 8% sugar, 6% soybean oil, and 1% cholesterol (503 kcal per 100 g feed) [27]. The body weight of the mice was measured at the end of the modeling time (8 weeks). After 8 weeks, blood samples were collected from the orbital sinus to test the serum total cholesterol (TC) to confirm that the high-fat diet (HFD) obese mice model was established.

#### 2.11.2. Administration of Animals and Treatment

After establishing the high-fat model, the successfully-modeled mice were divided into 4 groups, namely the high-fat model control (HC) group, GEN-treated (GT) group, GEN SD (1:7)-treated (ST) group, and GEN PM (1:7)-treated (PT) group. Including the NC group, the study was conducted on 5 groups of animals. The treatments of the ST group and PT group, equivalent to a 40 mg/kg dose of GEN, were suspended in peanut oil, and the treatments were administered to the mice through oral gavage. Body weights were measured weekly. At the end of the experiment (12 weeks), all mice were fasted for 12 h to eliminate the influence of food, and then were anesthetized by an intraperitoneal injection of sodium pentobarbital. Blood samples were drawn from the orbital sinus. The serum was immediately prepared from the plasma and was stored at −80 °C. The serum TC and triglyceride (TG) levels were measured using the enzymatic colorimetric method (UV-) with commercial assay kits (Nanjing Jiancheng Institute of Bioengineering, Nanjing, China) according to the manufacturer’s instructions. The liver, kidney, and epididymal adipose tissues were removed and quickly immersed in normal saline to remove blood, blotted with filter paper to remove the excess water on the surface, and then weighed and recorded. The liver tissues were fixed in 10% formaldehyde, embedded in paraffin, and sliced to determine the morphology by hematoxylin and eosin (H&E) staining. The liver index, kidney index, and adipose fat index were calculated [28,29].

### 2.12. Statistical Analysis

All data were analyzed using one-way analysis of variance (ANOVA) to compare whether there were significant differences between groups, and then Duncan’s multiple comparison analysis was used. Data analyses were carried out with SPSS 20.0 software (IBM Inc., Armonk, NY, USA). *p* ≤ 0.05 was considered statistically significant.

## 3. Results and Discussion

### 3.1. Characterization of Prepared Samples

**Figure 2:**
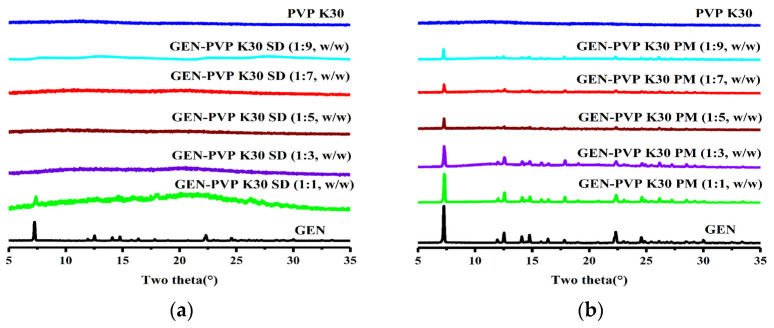
PXRD patterns of GEN, PVP K30, and (**a**) SDs/ (**b**) PMs of GEN:PVP K30 in ratios of 1:1 to 1:9.

PXRD was used to determine the absence of crystalline GEN. Except for the 1:1 ratio, the characteristic PXRD peaks of GEN could not be observed in any ratio powders but were clearly observed in all PMs (Figure 2). These results indicate that GEN exists in an amorphous state in all the SD powders except sample (1:1).

**Figure 3:**
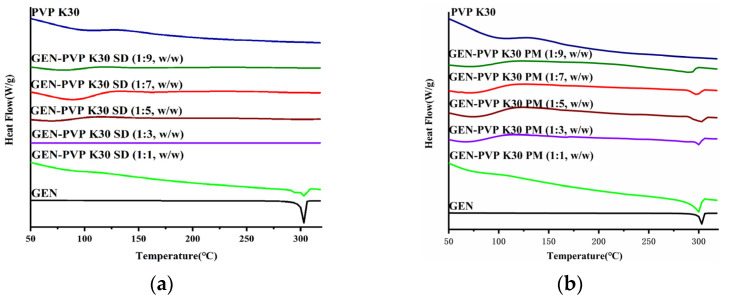
DSC patterns of GEN, PVP K30, and (**a**) SDs/ (**b**) PMs of GEN:PVP K30 in ratios of 1:1 to 1:9.

The DSC thermal behaviors of the GEN, PVP K30, SD (1:1–9), and PM (1:1–9) samples are shown in Figure 3. Pure GEN exhibited a sharp-melting endotherm at 302 °C, confirming the crystalline form [30]. PVP K30 displayed a broad endothermic peak between 75 and 135 °C, demonstrating a loss of water from the hygroscopic polymer [31]. The PMs in all five ratios exhibited a low intensity melting endotherm, which could be due to the polymer dilution effect [32]. No endothermic peaks were observed in any ratio powders except for the 1:1 ratio, implying that GEN had been incorporated in an amorphous form, consistent with the PXRD results.

### 3.2. Drug Loading Efficiency of SDs

According to Table 1, the practical drug loading efficiency of SD (1:3) and SD (1:5) differed greatly from the theoretical drug efficiency. However, the practical drug loading efficiency of SD (1:7) and SD (1:9) was basically equal with the theoretical drug efficiency.

### 3.3. Saturated Solubility and In Vitro Dissolution Testing

According to Table 2, compared with GEN, the saturated solubility of SDs was improved. The efficiency of solubilization was as follows: SD (1:9) > SD (1:7) > SD (1:5) > SD (1:3). It was found that the solubility of SDs increased when the proportion of the PVPK30 increased. A higher amount of the hydrophilic carrier present in the surrounding of the drugs will further enhance the wettability and support saturated solubility of the drug.

**Figure 4:**
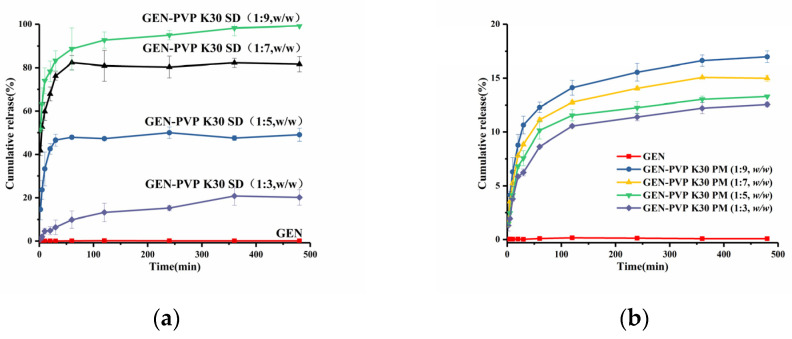
Dissolution profiles of GEN and (**a**) SDs/ (**b**) PMs of GEN:PVP K30 in ratios of 1:3 to 1:9.

The result of the in vitro GEN dissolution of the SD and PM samples in pH 6.8 are shown in Figure 4. The release of GEN within 480 min is only 0.17%. The GEN release from PM- (1:3, 1:5, 1:7 and 1:9) reaches 12, 13, 14, and 16%, respectively (Figure 4b). This may be due to the surface activity and crystallization inhibition of PVP K30, which can, to some extent, slow down the crystallization of GEN, thus maintaining the supersaturation of the drug [33].

The GEN release from SD (1:3) tends to equilibrate at 20% after 360 min, from SD (1:5) at 46% after 30 min, from SD (1:7) at 82% after 60 min, and from SD (1:9) at 99% after 480 min (Figure 4a). At 60 min, the drug release of SD (1:7) increased by 482 times compared to the GEN raw material. Erizal et al. developed an amorphous SD with PVP K30 by a solvent co-evaporation method, but the drug release of GEN-PVP K30 (1:2) SD increased by only 4.36 times after 60 min [34]. The cumulative solubility increases for SD with an increasing polymer carrier ratio. This may be since PVP K30 is a hydrophilic polymer that forms a concentrated polymer layer on the drug dissolving surface. The drug must pass through the polymer layer before being released into the solution medium [35]. Two modes of drug release have been proposed, namely carrier-controlled and drug-controlled.

In a carrier-controlled release, the drug particles are well dispersed in the polymer layer and diffuse into the solution medium as solvated molecules. In a drug-controlled release, the drug is not completely dissolved in the polymer layer and is released into the solution medium as solid crystalline particles [36]. In a drug-controlled release, the drug solubility is determined by the nature of the drug itself, while for a carrier-controlled release, it is determined by the nature of the carrier. Both types of drug release may operate simultaneously [37]. Thus, as the carrier ratio increases, the thickness of the polymer layer increases, the drug is better dispersed, and the SD amorphous drug is better stabilized and dissolved in the polymer layer. Molecular diffusion into the solution is favored by the higher solubility of the amorphous solid form, leading to a concentration in excess of the saturation solubility of crystalline GEN. In addition, when the proportion of the carrier increases, the viscosity of the SD in the solution increases, thus reducing the aggregation of drug particles and inhibiting drug crystallization [38]. Therefore, it can maintain the supersaturation of the drug during dissolution for a longer period of time and improve the cumulative solubility.

These results demonstrate that the SD improved the saturation solubility and dissolution rate of GEN compared with pure GEN and the PM.

However, SD (1:9) has a lower drug load since the amount of carrier PVP K30 is proportionally large and the preparation process takes a long time. Therefore, after consideration, SD (1:7) was selected for subsequent pharmacokinetic and pharmacodynamic studies.

### 3.4. Structure of GEN SD

**Figure 5:**
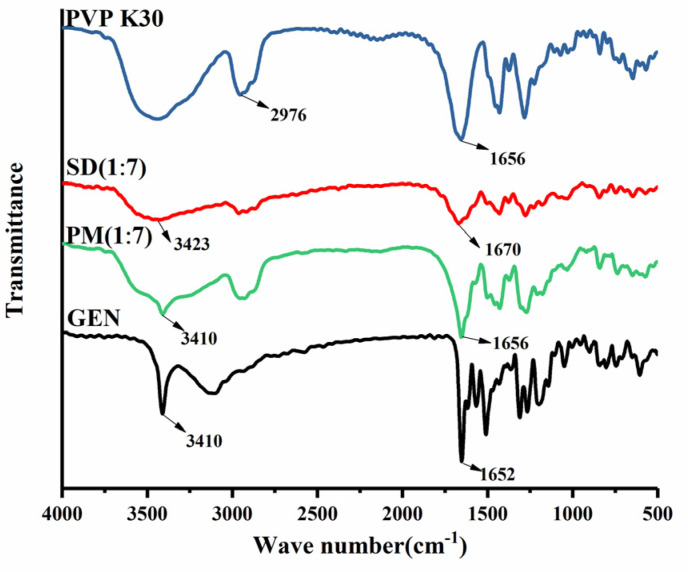
FT-IR spectra of GEN, PVP K30, and SD/PM of GEN:PVP K30 in a ratio of 1:7.

The FT-IR spectra of GEN, PVP K30, SD (1:7), and PM (1:7) are shown in Figure 5. The stretching vibration of the O-H and C=O groups of GEN appear at 3410 and 1652 cm^−1^, respectively [1]. PVP K30 shows C-H and C=O stretches at 2976 and 1656 cm^−1^, respectively. The carbonyl group of PVP K30 tends to hydrogen bond with favorable functional groups of drugs, usually leading to bathochromic shifting or the broadening of the peaks [39]. In the spectrum of SD (1:7), the stretching vibration of the O-H of GEN and the C=O of PVP K30 disappears at 3410 cm^−1^ and 1656 cm^−1^, respectively. Meanwhile, broad bands at 3423 and 1670 cm^−1^ attributed to the intermolecular hydrogen bonding between GEN and PVP K30 appeared. In PM (1:7), the FT-IR spectrum did not show significant alterations to the characteristic peaks of GEN, indicating the absence of a molecular interaction between GEN and PVP K30.

### 3.5. Morphology Evaluation

**Figure 6:**
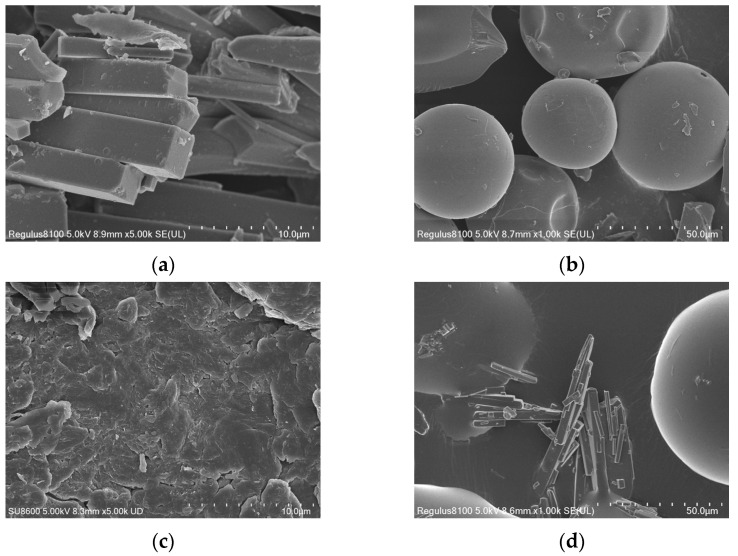
SEM images of (**a**) GEN, (**b**) PVP K30, and (**c**) SD/ (**d**) PM of GEN:PVP K30 in a ratio of 1:7.

The morphological characteristics of GEN, PVP K30, SD (1:7), and PM (1:7) are shown in Figure 6. A scanning electron microscope can visually observe the surface morphology of the material. The image revealed that raw GEN has a well-defined prism-shaped crystal structure (Figure 6a) [1]. PVP K30 occurred as spherical particles that were irregularly sized (Figure 6b), and these two particles formed can coexist in a simply mixed formation in the PM (1:7) (Figure 6d). However, GEN crystals were not observed in the SD (1:7) (Figure 6c), but showed irregular lumps that were different from the morphology of raw GEN and PVP K30 [40].

### 3.6. Stability Studies

**Figure 7:**
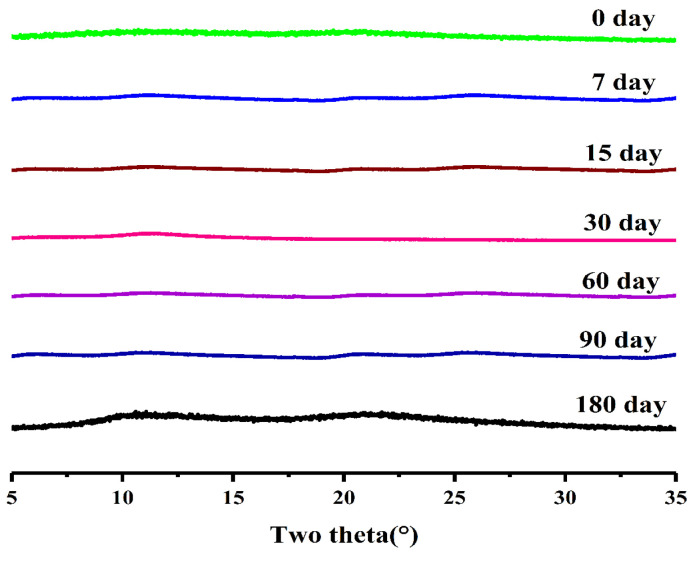
PXRD patterns of SD (1:7) with time under stability conditions.

The drug in the SD exists in an amorphous state with high energy, tending to convert into a more stable crystalline state with time [41]. It has been shown that the strong hydrophobic drugs-PVP K30 hydrogen bonds can inhibit the hygroscopicity of PVP K30 to a certain extent, thus maintaining the stability of SDs [42]. Therefore, PXRD was used to observe the stability of SDs. The results in Figure 7 showed that no crystal diffraction peaks appeared in the SDs at different times, proving that SD (1:7) was stable at 40 °C /75% RH.

### 3.7. In Vivo Pharmacokinetics

**Figure 8:**
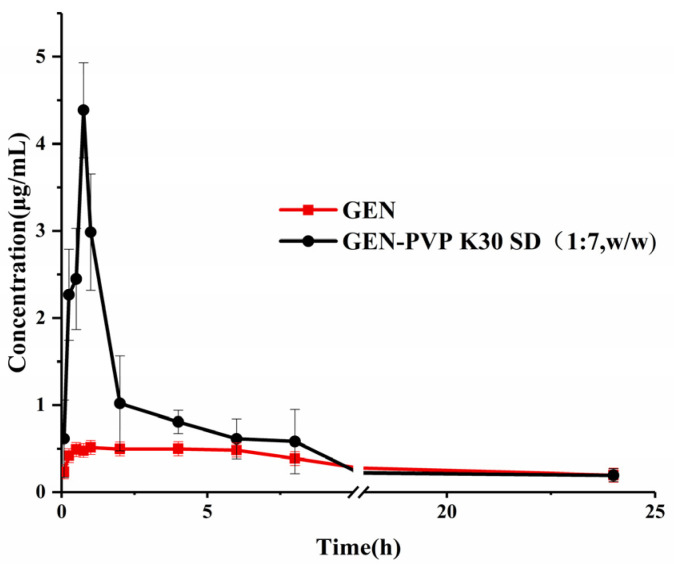
Plasma concentration–time profiles of GEN and SD (1:7) after oral administration.

The mean plasma concentration–time profiles and the pharmacokinetic parameters after the oral administration of GEN and SD (1:7) in SD rats are present in Figure 8 and Table 3. The C_max_ value of the SD (1:7) group was 4.4 ± 0.5 μg/mL, whereas that of the GEN group was 0.6 ± 0.1 μg/mL. This finding indicates that SD (1:7) could significantly increase oral GEN absorption compared with pure GEN. The GEN in SD was highly dispersed in the soluble carrier PVP K30. Because of its high dispersion, the drug was easier to dissolve and release in vitro. SD can induce super-saturated drug dissolution and enhance absorption. Therefore, after oral administration, the SD will dissolve in the intestinal segment and be absorbed by the gastrointestinal tract in the form of a solution, thus improving bioavailability [43]. The AUC_0–24_ of SD (1:7) (10.7 ± 1.6 µg/mL·h) was higher than that of GEN (5.2 ± 0.8 µg/mL·h), and the bioavailability of GEN in the SD was 106% greater than that of pure GEN. Meanwhile, the T_max_ of SD (1:7) was only one sixth that of GEN (0.8 ± 0.1 and 4.7 ± 0.9 h, respectively). These results indicated that the SD accelerates the absorption of GEN and improves bioavailability consistent with the in vitro dissolution data.

### 3.8. Gen SD Supplementation to Reduce Obesity

**Figure 9:**
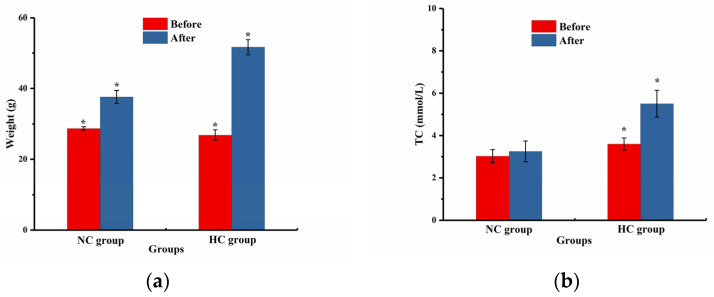
(**a**) Body weight and (**b**) TC level change before (NC group) and after (HC group) obesity modeling. (NC group: normal control group; HC group: high-fat model control group; * *p* < 0.05).

The changes in the TC level and body weight in the NC group and HC group before and after modeling are shown in Figure 9. After modeling, the TC level in the NC group mice did not change notably while the TC in the HC group mice changed significantly. At the end of the modeling, the average body weight of the HC group mice was >15% higher than that of the NC group mice, indicating the successful modeling of obese mice [44].

**Figure 10:**
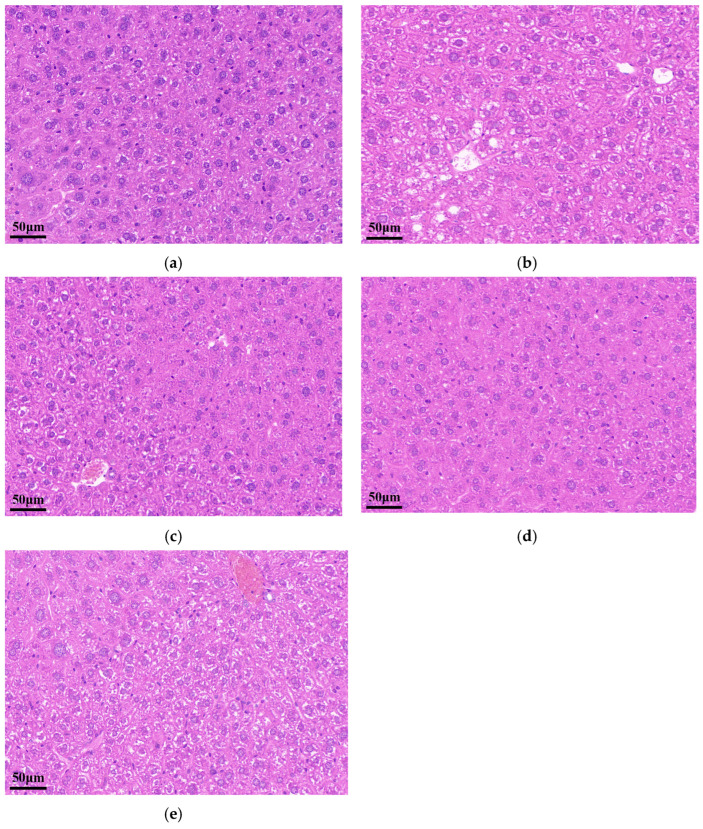
Liver histology with hematoxylin-eosin (H&E) staining. (**a**) NC group, (**b**) HC group, (**c**) GT group, (**d**) ST group, and (**e**) PT group. (NC group: normal control group; HC group: high-fat model control group; GT group: GEN-treated group; ST group: GEN SD (1:7)-treated group; PT group: GEN PM (1:7)-treated group; 200× magnification; Scar bar: 50 μm).

A long-term high-fat diet can induce obesity and promote lipid accumulation in the liver and kidneys [28,45,46] while increasing the size of fat cells in adipose tissue and the corresponding organ weight [47]. In addition, the weight of the adipose tissue around the perirenal and epididymal of the animal accounted for a certain percentage of the total adipose tissue weight [29]. Therefore, the ratio of the weight of the liver, kidney, perirenal, and epididymal adipose tissue to the body weight of the mice was selected to reflect the obesity status. The liver index, kidney index, adipose fat index, body weight, and liver histological observation are shown in Table 4 and Figure 10. It is clear from the results that the HC group mice had a significantly higher lipid ratio and body weight than the NC group mice. The adipose fat index and body weight of the ST group were significantly lower than those of the GT group and PT group (*p* < 0.05). In addition, the kidney index of the ST group was lower than that of the GT group, although there was no significant difference. The ST group exhibited a lower liver index and kidney index than those of the PT group, but no significant difference was observed. The ST group showed the best effect on the reduction of lipid accumulation compared to the GT group and PT group. In a histological liver analysis by H&E staining, compared with the NC group, the HC group had enlarged hepatocytes and presented round vesicles indicative of significant lipid accumulation. However, the GT group, ST group, and PT group significantly alleviated hepatic lipid accumulation. These results demonstrate that GEN treatment can reduce lipid accumulation and control body weight and, moreover, that SD (1:7) can enhance the effect of GEN on fat accumulation in HFD-induced obese mice.

**Figure 11:**
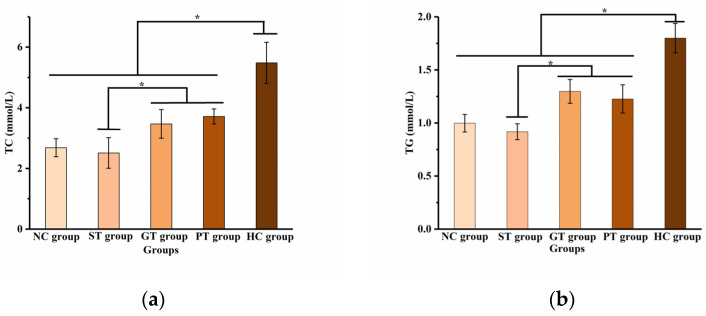
Effect of GEN on the levels of TC (**a**) and TG (**b**) in serum of different groups. (NC group: normal control group; HC group: high-fat model control group; GT group: GEN-treated group; ST group: GEN SD (1:7)-treated group; PT group: GEN PM (1:7)-treated group; TC: total cholesterol; TG: triglycerides; *n* = 10 per group; * indicates significant difference between groups, *p* < 0.05).

Obesity leads to dyslipidemia, usually in the form of high serum levels of TC and TG [48]. As can be seen in Figure 11, the TC and TG levels were significantly higher in the HC group than in the NC group. Meanwhile, the TC and TG levels of the GT group, ST group, and PT group were significantly lower than the HC group (*p* < 0.05). Furthermore, the TC and TG levels of the ST group were significantly lower than those of the GT group and PT group (*p* < 0.05). This suggests that SDs improve the ability of GEN to lower blood lipids.

## 4. Conclusions

In the present study, an SD of GEN with PVP K30 as the carrier has been successfully prepared using a solvent rotary evaporation method. The SDs can improve the cumulative dissolution extent and rate of GEN and enhance its oral bioavailability. Moreover, the SDs improve the ability of GEN to control obesity symptoms. Thus, the potential for clinical applications has been demonstrated for GEN SDs in treating lipid metabolism disorders to improve obesity control.

## Figures and Tables

**Table 1:** The practical drug loading efficiency of all the SDs.

Weight Ratio of GEN and PVP K30	Theoretical Drug Loading Efficiency of SDs (%)	Practical Drug Loading Efficiency of SDs (%)
1:3	25.0	19.0 ± 0.4
1:5	16.7	13.5 ± 0.7
1:7	12.5	11.0 ± 0.4
1:9	10.0	9.2 ± 0.6

**Table 2:** Saturated solubility of GEN and all the SDs.

	Solubility (µg/mL)	Fold
GEN	1.8 ± 0.1	-
SD (1:3)	141.3 ± 8.5	78.5
SD (1:5)	768.1 ± 107.9	426.7
SD (1:7)	1846.6 ± 97.8	1025.9
SD (1:9)	3981.5 ± 139.6	2211.9

**Table 3:** Main pharmacokinetic parameters of GEN and SD (1:7) after a single 50 mg/kg oral dose.

Pharmacokinetic Parameter	GEN	SD (1:7)
AUC_0~24_ (µg/mL·h)	5.2 ± 0.8	10.7 ± 1.6 *
C_max_ (μg/mL)	0.6 ± 0.1	4.4 ± 0.5 *
T_max_ (h)	4.7 ± 0.9	0.8 ± 0.1 *
T_1/2_ (h)	6.7 ± 0.2	4.5 ± 1.4

Notes: Each value is the mean ± standard error, *n* = 5. * Represents the significant difference at *p* < 0.05 vs. GEN.

**Table 4:** Effect of GEN, SD (1:7), and PM (1:7) on the levels of liver index, kidney index, adipose fat index, and body weight in HFD-induced obesity mice.

Groups	Liver Index	Kidney Index	Adipose Fat Index	Body Weight
NC group	4.4 ± 0.3	1.3 ± 0.1	1.6 ± 0.4 ^+^*	43.7 ± 1.9 *
HC group	5.2 ± 0.4	1.6 ± 0.1	6.9 ± 1.6 ^+^	53.2 ± 2.7
GT group	4.5 ± 0.5 *	1.4 ± 0.1 *	4.7 ± 1.6 *^+^	45.2 ± 2.8 *^+^
ST group	4.5 ± 0.2 *	1.3 ± 0.1 *	3.8 ±1.3 *	44.6 ± 2.0 *
PT group	4.6 ± 0.4	1.5 ± 0.1 *	4.6 ± 0.8 ^+^	47.0 ± 2.0 *^+^

Notes: NC group: normal control group; HC group: high-fat model control group; GT group: GEN-treated group; ST group: GEN SD (1:7)-treated group; PT group: GEN PM (1:7)-treated group; each value is the mean ± standard error, *n* = 10 per group. * Represents the significant difference at *p* < 0.05 vs. MG. + Represents the significant difference at *p* < 0.05 vs. SG.

## Data Availability

The data can be shared up on request.
